# Factors associated with advanced-stage diagnosis of breast cancer in north-west Ethiopia: a cross-sectional study

**DOI:** 10.3332/ecancer.2021.1214

**Published:** 2021-03-25

**Authors:** Aragaw Tesfaw, Mulu Tiruneh, Tadese Tamire, Tewodros Yosef

**Affiliations:** 1 Debre Tabor University, College of Health Sciences, Department of Public Health, PO Box 272, Debre Tabor, Ethiopia; 2 Debre Tabor University, College of Health Sciences, Department of Anesthesia, PO Box 272, Debre Tabor, Ethiopia; 3 Mizan-Tepi University, Department of Epidemiology and Biostatistics, School of Public Health, College of Medicine and Health Sciences, PO Box 260, Mizan-Aman, Ethiopia

**Keywords:** breast cancer, advanced stage, determinants, Ethiopia

## Abstract

**Background:**

Breast cancer tumours are the most common malignant tumours among women in Ethiopia. Although advanced-stage diagnosis of breast cancer is a common problem, evidence-based information is lacking about the magnitude and determinants of advanced-stage presentation in north-west Ethiopia.

**Methods:**

An institution-based, cross-sectional study was conducted at the oncology units of the University of Gondar and Felege Hiwot specialised hospitals. Stages III and IV were considered advanced stage, whereas stages I and II were considered early stages. Data were collected prospectively on newly diagnosed breast cancer patients and entered using the EPI Info version 7.2 and analysed using Statistical Package for the Social Sciences version 23. Multivariable logistic regression was used to identify the determinants of advanced-stage diagnosis of breast cancer. A *p*-value < 0.05 was used as the cut-off point to select the determinants of the advanced stage.

**Result:**

About 71.2% of breast cancer patients presented with advanced-stage disease. The median age of patients was 40 years. Rural residence (adjusted odds ratio (AOR) = 1.7; 95% confidence interval (CI): 1.02, 2.96), painless breast lump/wound (AOR = 2.5; 95% CI: 1.45, 4.13), travel distance ≥5 km (AOR = 3.2; 95% CI: 1.72, 5.29), not practising breast self-examination (BSE) (AOR = 2.9; 95% CI: 1.30, 6.52), time to presentation ≥3 months (AOR = 1.4; 95% CI: 1.02, 2.37) and misdiagnosed at first visit (AOR = 1.9; 95% CI: 1.09, 3.59) were determinants of advanced-stage breast cancer.

**Conclusion:**

Nearly three-quarters of the patients were diagnosed with advanced-stage breast cancer. Not practising BSE, travel distance ≥5 km, rural residence, painless breast wound/lump and being misdiagnosed at first visit were important determinants of advanced-stage diagnosis of breast cancer. Focused awareness creation programmes for the public and increasing cancer diagnostic centres in the country are crucial to downstage breast cancer at presentation.

## Background

Breast cancer is the most common malignant tumour among women. It can be characterised by distinct clinical, pathological and molecular characteristics [1, 2]. It is a growing public health concern globally, as the leading cause of cancer with high mortality rates in low- and middle-income countries [3]. According to the World Health Organization, in 2015, around 60,000 new breast cancer cases were diagnosed in Ethiopia annually, although the country has shortages in trained cancer healthcare providers, diagnostic and treatment centres [4]. As evidence shows, the incidence of breast cancer cases in Ethiopia is growing at an alarming rate and it is now the most frequently diagnosed tumour among women with an estimated incidence rate of 43 cases per 100,000 women. According to the Addis Ababa population-based cancer registry report, breast cancer accounts for 33% of all female cancer cases and 23% of all cancers in the country [5, 6].

An important factor in the prognosis and survival of breast cancer is the early detection of cases. Breast self-examination (BSE), clinical breast examination and mammography are commonly used methods of early detection in the current era. BSE is the simplest, quickest and cost-free procedure for the early detection of breast cancer mainly in developing countries [7, 8].

Unless diagnosed and treated early, breast cancer is life-threatening. Advanced stage and large tumour size at diagnosis are associated with decreased survival. In Ethiopia, the majority of breast cancer patients are diagnosed at advanced stages and their survival is poor; as a result, most patients need palliative care [9]. A multicentre study in Africa found that nearly two-thirds (61%) of the breast cancer patients are diagnosed at advanced stage (stages III and IV) [10]. Several factors have been found to contribute to late-stage diagnosis of breast cancer. A systematic review of several published studies in Africa found that lack of information or knowledge, poor health facilities, negative symptom interpretation, fear, belief in alternative medicine, late screening or detection, lack of trust and access to healthcare were identified as factors delaying the presentation of breast cancer patients among African women [11].

Financial problems, ignorance and misinterpreting early symptoms of breast cancer were the major causes of late diagnosis in Sudan [12]. Similarly, in Uganda, the most commonly identified barriers to early diagnosis of breast cancer were lack of knowledge about early diagnosis, economic barriers to accessing care, fear and poor social support [13].

Studies also reported that patient and healthcare system delays affect the cancer stage at diagnosis, even though their strength is different in particular countries. Delays also occurred within the healthcare system, due to problems in the first and second levels of healthcare associated with prolonged waiting times to get appointments, accessibility barriers and quality of care [14, 15].

In Ethiopia, there is only one radiotherapy centre which is supposed to serve the entire national population but it is inaccessible to majority of the rural population. Even though the country has launched a national cancer control plan in recent years to tackle this problem, the main focus of the government and healthcare systems was towards infectious disease like malaria, HIV/AIDS and TB than cancer until recent years [16]. As a result, majority of the reported cases of cancer present with advanced disease and most of the patients have an incurable disease [6, 17].

The reasons for late presentation and advanced-stage diagnosis in most breast cancer patients might be due to little awareness, associated stigma, inadequate screening or diagnostic services and the lengthy process of referral to the oncology centre [18]. However, the magnitude and factors contributing to late-stage diagnosis of breast cancer remain a study area where strong evidence is lacking since some of the available reports are based on the only oncology and radiotherapy centre at Addis Ababa, which may not be representative of the rural population who are disadvantaged due to their distance to the oncology centre. Understanding the factors contributing to advanced-stage diagnosis of breast cancer in Ethiopia is essential for planning targeted interventions that help to mitigate premature death of women from breast cancer. It will also be helpful to alert the policymakers and national programmers about the need to develop effective interventions for addressing the patient and healthcare system challenges that hinder early diagnosis and optimal utilisation of services regarding breast cancer care. Therefore, this study aimed to identify the determinants of advanced-stage diagnosis of breast cancer at two oncology centres which particularly serve the majority of the rural population in the Amhara region, in the north-western part of Ethiopia.

## Methods

### Study design and setting

This study was a cross-sectional study conducted at two comprehensive specialised hospitals (Felege Hiwot and Gondar University hospital) in Amhara Region, Northwest Ethiopia. The study was conducted from 1 September 2019 to 30 April 2020. The hospitals are used as the only oncology referral centres for all cancer cases in the north-western corner of the country. Felege Hiwot comprehensive specialised hospital is found in Bahirdar (city in Amhara regional state), 565 km far from Addis Ababa (the capital city of Ethiopia). University of Gondar hospital is a comprehensive, specialised and teaching hospital found in Gondar town, 737 km far from the capital city of Ethiopia (Addis Ababa). The oncology unit of the hospitals currently provides diagnostic (fine needle aspiration cytology, biopsy, chest X-ray, ultrasound), imaging (CT scan and magnetic resonance imaging), surgical and chemotherapy treatment services for cancer patients including breast cancer patients. But there are no radiotherapy services for cancer patients until now. They are also used as teaching hospitals for the University of Gondar and Bahirdar University to train different healthcare professionals in different specialisations.

### Study population and data collection procedures

Patients with histologically confirmed breast cancer who were treated between 1 September 2019 and 30 April 2020 at the two hospitals were included in the study. Data were collected using interviewer-administered structured questionnaires which were developed from the reviewed literature [19–21]. The questionnaires consisted of socio-demographic characteristics, patient’s history of their diagnostic journey, medical and reproductive history and tumour characteristics.

Patients were asked about their breast cancer diagnosis journey from their first initial symptom recognition to their first healthcare contact. The data collectors were four nurses who are working in the oncology unit of the hospitals. Two supervisors (public health experts) were assigned for supervising the data collectors in each of the oncology units.

### Measurements

Time to presentation was defined as the time from a patient’s first detection of the first symptom/breast abnormality until their first healthcare facility visit and it was categorised as ≥3 months (long presentation delay ) and <3 months (short delay). Healthcare system delay time was defined as the time from the first healthcare visit until pathologically confirmed diagnosis of breast cancer. Total diagnostic delay time was defined as the time from first symptom recognition until final confirmed diagnosis (presentation delay plus healthcare system delay time) [19, 22]. Breast cancer staging was carried out using the American Joint Committee on Cancer (AJCC, 7th edition) cancer staging system (tumour, node and metastasis) [23]. Breast cancer stages III and IV were defined as advanced stages and patients diagnosed with breast cancer stages I and II were considered to be in the early stages [19, 20]. Travel distance was defined as an estimated distance from patients home to the nearby healthcare facilities.

### Data quality and analysis procedures

The quality of the data was assured by using a structured and pre-tested questionnaire. The questionnaire was prepared in a simple and easily understandable language which was initially prepared in English, and later translated to the local language (Amharic) to facilitate communication.

Before analysis, data were checked for completeness and internal consistency, then they were coded and entered using the EPI Info version 7.2 and analysed using Statistical Package for the Social Sciences version 23. Descriptive statistical analysis with chi-square test was used to present the socio-demographic and clinical characteristics of the patients. Binary and multivariable logistic regression analyses were used to identify determinants of advanced-stage diagnosis of breast cancer. An adjusted odds ratio (AOR) and 95% confidence interval (CI) were used to describe the final model. *p*-values less than 0.05 were used to select statistically significant results.

### Ethical consideration

An ethical clearance letter was obtained from the research ethics committee of the College of Health Science, Debre Tabor University. The study hospitals were informed through a support letter. Informed verbal consent was obtained from all breast cancer patients who participated in the study. Confidentiality of information and privacy of participants during the interview was respected. A detailed explanation was given to patients about the objectives and benefits of the study.

## Result

### Socio-demographic characteristics of patients

The median age of breast cancer patients at diagnosis was 40 years (range: 20-87 years). More than two-thirds (249, 67.1%) of the breast cancer patients were from rural areas. A higher proportion of rural women (188, 75.5%) had a significantly advanced stage at diagnosis compared to urban women (76, 62.3%) (*p* = 0.008**)**. Majority of the women (173, 83.2%) who travelled more than 5 km to arrive at the nearby healthcare facility were diagnosed at an advanced stage compared to women who travelled less than 5 km (91, 55.8%) (*p* < 0.001) (Table 1).

### Clinical characteristics of breast cancer patients

More than three-quarters of the patients (320, 86.3%) presented with a tumour which involved positive axillary lymph node. More than half (209, 56.3%) of the patients had a tumour size of more than 5 cm. The median tumour size at diagnosis was 6 cm (range: 2-17 cm). Almost all patients (347, 93.5%) had invasive ductal carcinoma. Lobular carcinoma was found in 18 (4.9%) patients. About six (1.6%) breast cancer cases were mixed types of tumours and inflammatory carcinomas. The result showed that 140 (45.6%) patients had poorly differentiated invasive carcinoma (grade III tumour) and 79 (40.7%) had moderately differentiated invasive carcinoma (grade II tumour).

Among the breast cancer patients, the majority (209, 56.3%) were premenopausal women. A total of 74 (19.9%) had a family history of breast cancer and 11 (3%) patients were HIV-positive. Among the patients, 142 (38.3%) had a history of comorbidities. More than three-quarters (328, 88.4%) of the breast cancer patients had breast lumps and 161 (43.4%) had painful breast wounds or ulcers at presentation (Table 2).

### Magnitude of diagnostic delay and advanced-stage diagnosis

The overall magnitude of diagnostic delay (time from first symptom recognition until confirmed diagnosis of breast cancer ≥3 months) was 295 days, while the time to first healthcare contact (presentation delay ≥3 months) was 281 days and healthcare system delay time (first healthcare contact until final pathological confirmed diagnosis of cancer above the average time/healthcare system delay) was 190 days. The proportion of an advanced stage diagnosis was 71.2%, 95% CI (67.5%–77.1%). Among these, 211 (56.9%) patients were diagnosed at stage III and the remaining 53 (14.3%) were diagnosed at stage IV. Stages I and II account for 37 (10%) and 70 (18.9%) breast cancer cases, respectively. The average time taken from first medical visit until confirmed diagnosis of breast cancer (healthcare system delay time) was 39 days with a range of 7-276 days. The average patient presentation delay time was 8 months with a range of 2 weeks to 3 years. A significantly higher proportion of patients (211, 75.1%) who had a long patient presentation delay of more than 3 months were diagnosed at advanced stage (stages III and IV) than those who had short delay (*p* = 0.003).

The most common reasons mentioned for late presentation to healthcare facilities were lack of awareness about early symptoms of breast cancer (345, 92.9%), use of traditional and spiritual treatment options (286, 77.1%) and financial problems (217, 58.5%). About 138 (37.2%) breast cancer patients had a history of breast problems before the current condition and 45 women (12.1%) practised BSE. Women who did not practise BSE (249, 76.4%) presented with advanced stages than their counterparts (15, 33.3%) (*p* < 0.0001). More than two-thirds of the breast cancer patients (256, 69.0%) had a history of using traditional treatments. A significantly higher proportion of women (200, 78.1%) who used traditional treatments were diagnosed at an advanced stage as compared with those who did not use any traditional treatments (64, 55.7%) (*p* < 0.001).

### Determinants of advanced-stage diagnosis among breast cancer patients

Patients who were from a rural residence were 1.7 times more likely to be diagnosed at an advanced stage than those from an urban residence (AOR = 1.7; 95% CI: (1.02, 2.96)). Patients who presented with painful breast lumps/wounds were approximately 2.5 times more likely to be at an advanced stage than patients who had painless wounds/lumps at presentation (AOR = 2.5; 95% CI: (1.45, 4.127)). Travel distance to the nearby healthcare facility ≥5 km was also associated with advanced-stage diagnosis of patients with breast cancer (AOR = 3.2; 95% CI: (1.72, 5.29)). Women who did not practise BSE were approximately three times more likely to be diagnosed at an advanced stage (AOR = 2.9; 95% CI: (1.30, 6.52)). In addition, women who delayed more than 3 months to present at a healthcare facility were 1.4 times more likely to be diagnosed at advanced stage (AOR = 1.4; 95% CI: (1.02, 2.37)). Women who were misdiagnosed at their initial visit were 1.9 times more likely to have advanced-stage breast cancer at diagnosis than women who did not have a history of misdiagnosis (AOR = 1.9; 95% CI: (1.09, 3.59)) (Table 3).

## Discussion

Advanced stage at diagnosis is a common problem in cancer care and has been widely reported, which significantly affects breast cancer survival globally. Most breast cancer patients in developing countries suffer very long delays and are diagnosed at advanced stages and have high mortality rates [21, 24, 25]. Several studies classify factors contributing to advanced-stage diagnosis into patient-mediated factors and healthcare system-related factors [19, 20, 26].

Our study found the magnitude of advanced-stage diagnosis to be 71.2%, 95% CI (67.5%–77.1%), from which stage III accounts for 56.9% and stage IV accounts for 14.3% of the cases. The result is in line with a study finding in Libya (54.0% for stage III and 11.5% for stage IV disease, respectively) [22]. However, this finding is much higher than study findings from northern Pakistan in which only 39% of the patients presented with late-stage breast cancer [27] and the prevalence of locally advanced and metastatic disease at presentation was 47% in Mexico [28]. Our finding is much higher when compared with the study conducted in Western Cape Province (51%) and Soweto (22%) in South Africa [29, 30]. But the study is lower than the study findings from Tanzania (63% for stage III and 21.4% for stage IV) [31] and Nigeria (157 with stage III (52.4%) and 46 (15.3%) with stage IV) [21]. The difference might be due to variations in sample size and socio-economic differences between the countries.

Breast cancer patients in this study experienced a median total diagnostic delay of 186 days. The average healthcare system and patient delay were 39 and 233 days, respectively. This study’s findings are lower than studies conducted in Mexico (median total delay of 270 days) [32] and Rwanda [19], but higher than studies conducted in Morocco (median total diagnostic delay of 120 days, patient delay (65 days), healthcare system delay (median = 50 days)) [33] and Nigeria in which presentation delay and diagnostic delay were 35.3% and 30.5%, respectively [34]. The variations might be due to a difference in sample population characteristics or other sociocultural and infrastructure differences. The longer delay time might be explained as due to the low awareness of women about breast cancer symptoms and early detection methods as other sub-Saharan African countries in which lack of knowledge was the major contributor for late diagnosis, since most women did not recognise their early breast abnormalities as well as lack of trust in the medical care and preference to use spiritual and traditional treatment options than modern care [20, 24].

After controlling for the effects of potential confounders, the multivariable analysis of this study confirmed that presentation delay of more than 3 months, travel distance to the nearby healthcare facility ≥5 km, being from rural residence, not practising BSE, painful breast lumps/wounds and misdiagnosis at initial visit were important significant determinants of advanced-stage diagnosis of breast cancer. Our study showed the independent effects of presentation delay of more than 3 months on advanced-stage diagnosis of breast cancer. As the results show, the longer the patient presentation delay time, the more likely the patient diagnosed at an advanced stage of breast cancer*.* Patients who had a long presentation delay of more than 3 months were 1.4 times more likely to be diagnosed at late stage than those who had short presentation delay. This might be because the delay time may give enough time for progression and growth of the disease to advanced stages. A similar finding is reported in studies conducted in Rwanda [19], Senegal [26], Iran [35] and India [36] in which the longer delay time is a significant contributor for advanced-stage diagnosis of breast cancer.

In our study, the most common reasons for late presentation to medical care were lack of awareness about early symptoms of breast cancer, use of traditional and spiritual treatment options and financial problems. These reasons were reported in other similar studies in which poor economic status, illiteracy, lack of access to healthcare facility and negligence by patients or their family members, anxiety, fear, the necessity to prioritise immediate needs of daily life, non-disclosure of the situation and the view of medical care were the factors mentioned for presentation delay [22, 36, 37]. Low education, religious beliefs and patients who saw a traditional healer first were significantly associated factors for patient presentation delays [19, 38]. Similarly, women’s understanding about breast cancer symptoms (considering initial symptoms) as not serious until a sensory symptom appears, psycho-cultural and social factors were contributors for their late-stage diagnosis in Ghanaian women [39].

Women who were from rural areas were 1.7 times more likely to be diagnosed at advanced stage than urban women. The finding is also similar to a study in which women from rural area presented late to medical care compared to urban residences [19, 40, 41]. Distance to the nearby healthcare facility ≥5 km was also associated with late presentation of patients with breast cancer. Women living in the rural areas presented with advanced metastatic disease than women living in urban areas in Sudan and sub-Saharan Africa [42, 43]. This might be due to the long distance travelling to cancer diagnostic centres as well as the difficulty of getting information access about breast cancer to understand the early symptoms of the disease.

Breast cancer awareness and knowledge of the importance of early detection and diagnosis are poor in sub-Saharan African countries [20, 40]. Similarly, in our study, illiterate women were approximately 2.5 times more likely to delay presentation than those who had a secondary educational level and above. This finding is similar to other studies conducted in different countries [19, 22]. This could be explained as illiterate women will have low awareness about the disease, as well as their medical care seeking behaviour will be low.

Patients who had been presented with painful breast wounds were more likely to be diagnosed at advanced stage than patients who had painless wounds/lumps at presentation. This is because patients usually seek medical care when the early symptoms become painful and severe, which means after the disease is advanced and metastasised. Women who did not practise BSE were approximately three times more likely to diagnose at advanced stages. In Egypt, knowledge of BSE increased the likelihood of women to present in early stages [37]. Similarly, women who were able to perform BSE were diagnosed earlier compared to the others [40]. However, in our study, most of the patients did not practise BSE and as a result most of them detected their breast problems accidentally when they were washing their bodies, when they took off their clothes, when they breast feed and when the disease produced a discharge or pain. Only few (11.6%) breast cancer patients detected the abnormalities on their breast by BSE. This finding is similar to breast cancer patients in Libya, who noted lumps as an accidental finding, while four (2%) patients detected lump(s) during BSE [22]. A study conducted in 2011 among breast cancer patients in Ethiopia also revealed that 47.8% of the study participants knew nothing about breast cancer and never heard of the disease at all [44].

This study revealed that patients from rural areas were 1.7 times more likely to be diagnosed at late stage than those from an urban area. This finding is consistent with a study conducted in north Pakistan [27] and Sudan [42]. Travel distance to the nearby healthcare facility ≥5 km was also associated with advanced-stage diagnosis of patients with breast cancer. This finding could be explained by the fact that patients from rural areas are farther away from diagnostic healthcare facilities and have low levels of information about breast cancer and transportation access; as a result, they present after the disease is advanced and metastasised. This result is in line with a study carried out in sub-Saharan Africa in which distance to the diagnostic/treatment facility is a problem in most African countries [43].

Our study found that most of the breast cancer patients had a breast lump at presentation and the majority (242, 73.8%) of women who had a breast lump at presentation had advanced stage at diagnosis than their counterparts (*p* **<** 0.0001). This might be because patients may not consider their initial symptoms to be severe and they leave it until it becomes painful or women may relate the symptoms with other benign medical conditions and may think of it as self-limiting. Thus, most patients often first use traditional medicine and holy water before they accessed medical care and presented at an advanced stage of the disease. This finding is consistent with reports from other studies carried out in Africa in which women preferred the use of alternative medicine for long periods before seeking medical care [45].

Women who were misdiagnosed at their initial visit were 1.9 times more likely to have advanced-stage breast cancer at diagnosis than women who did not have history of misdiagnosis. Similarly, evidences showed that not only patient factors but also healthcare provider factors contribute to the delay in diagnosis and consequently advanced-stage presentation due to inadequate patient examination, use of inappropriate tests or miss interpretation of test results or relating symptoms to a healthcare problem other than cancer [46, 47].

### Strengths of the study

Based on the researchers’ knowledge, this study is the first study conducted in north-west Ethiopia to determine the factors associated with advanced stage at diagnosis of breast cancer. In addition, the study hospitals are the only oncology centres for all cancer patients in the north-western part of the country, so the representativeness of the data is very high since almost all cases of cancer are referred to these hospitals from primary healthcare units and district hospitals for diagnosis and for treatment follow-up.

### Limitations of the study

Although we used a cross-sectional study design, it is difficult to show the temporal relationship between the factors and the outcome; as a result, the study could not show the cause-and-effect relationship. In addition, our study is also affected by recall bias, since some patients faced difficulties to remember exact dates when their breast symptoms started and the dates in their diagnostic journey.

## Conclusion

This study found that more than two-thirds of the breast cancer patients in north-west Ethiopia experience diagnostic delay and advanced-stage diagnosis. Not practising BSE, travel distance ≥5 km, being at a rural residence, having a painful breast wound and misdiagnosed at first visit were important determinant factors for advanced-stage diagnosis of breast cancer. Therefore, focused awareness creation programmes should be implemented to downstage breast cancer at presentation at both public and healthcare system levels.

## Abbreviations and acronyms

BSE, Breast self-examination

## Declarations

### Ethics approval and consent to participate

An ethical clearance letter was obtained from a research ethics committee of the College of Health Science, Debre Tabor University. The study hospitals were informed through a support letter. Informed verbal consent was obtained from all breast cancer patients who participated in the study. Confidentiality of information and privacy of participants during the interview was respected. A detailed explanation was given to the patients about the objectives and benefits of the study.

### Consent for publication

Not applicable.

### Availability of data and materials

All the necessary data are available in the main manuscript document and its supporting information file.

### Competing interests

The authors declare that they have no competing interests.

### Funding

Not applicable.

### Authors’ contributions

AT was involved in the initiation of the idea, write-up of the proposal, data collection, data entry, data analysis and final manuscript write-up. MT was involved in data collection, data analysis and final manuscript write-up. TT and TY were involved in the final manuscript editing and write-up. All authors were involved in the approval of the final manuscript.

## Figures and Tables

**Table 1.** Socio-demographic characteristics of patients diagnosed at two comprehensive specialised hospitals in north-west Ethiopia, 2020.

Characteristics	Frequency (*n* (%))	Early stage (*n* (%))	Advanced stage (*n* (%))	value	*p*-value
**Age group**					
<30	55 (14.8%)	21 (38.2%)	34 (61.8%)	4.2	0.369
30–39	118 (31.8%)	33 (28.0%)	85 (72.0%)		
40–49	92 (24.8%)	21 (22.8%)	71 (77.2%)		
50–9	53 (14.3%)	15 (28.3%)	38 (71.7%)		
≥60	53 (14.3%)	17 (32.1%)	36 (67.9%)		
**Home residence**					
Rural	249 (67.1%)	61 (24.5%)	188 (75.5%)	6.9	0.008
Urban	122 (32.9%)	46 (37.7%)	76 (62.3%)		
**Marital status**					
Married	298 (80.3%)	89 (29.9%)	209 (70.1%)	0.7	0.379
Single	73 (19.7%)	18 (24.7%)	55 (75.3%)		
**Religion**					
Orthodox	172 (46.4%)	50 (29.1%)	122 (70.9%)	17.2	0.001
Muslim	96 (25.9%)	21 (21.9%)	75 (78.1%)		
Protestant	97 (26.1%)	30 (30.9%)	67 (69.1%)		
Catholic	6 (1.6%)	6 (100.0%)	0 (0.0%)		
**Educational status**					
Illiterate	136 (36.7%)	40 (29.4%)	96 (70.6%)	0.3	0.960
Primary education	153 (41.2%)	42 (27.5%)	111 (72.5%)		
Secondary education and above	82 (22.1%)	25 (30.5%)	57 (69.5%)		
**Occupational status**					
Housewife	215 (58%)	60 (27.9%)	155 (72.1%)	0.8	0.851
Farmer	94 (25.3%)	27 (28.7%)	67 (71.3%)		
Government employee	52 (14%)	16 (30.8%)	36 (69.2%)		
Other	10 (2.7%)	4 (40.0%)	6 (60.0%)		
**Distance to the healthcare facility**					
<5 km	163 (43.9%)	72 (44.2%)	91 (55.8%)	32.3	<0.001
≥5 km	208 (56.1%)	35 (16.8%)	173 (83.2%)		

**Table 2.** Clinical characteristics of patients at two comprehensive specialised hospitals in north-west Ethiopia, 2020.

Characteristics	Frequency (*n* (%))	Early stage (*n* (%))	Advanced stage (*n* (%))	value	*p*-value
**Menopausal status**					
Pre-menopausal	209 (56.3%)	69 (33.0%)	140 (67.0%)	4.6	0.098
Menopausal	69 (18.6%)	14 (20.3%)	55 (79.7%)		
Post-menopausal	93 (25.1%)	24 (25.8%)	69 (74.2%)		
**Family history of breast cancer**					
Yes	74 (19.9%)	25 (33.8%)	49 (66.2%)	1.1	0.294
No	297 (80.1%)	82 (27.6% )	215 (72.4%)		
**History of any breast problem before**					
Yes	138 (37.2%)	42 (30.4%)	96 (69.6%)	0.3	0.602
No	233 (62.8%)	65 (27.9%)	168 (72.1%)		
**Practising BSE**					
Yes	45 (12.1%)	30 (66.7%)	15 (33.3%)	35.7	**<0.0001**
No	326 (87.9%)	77 (23.6%)	249 (76.4%)		
**History of any comorbidities**					
Yes	142 (38.3%)	33 (23.2%)	109 (76.8%)	3.5	0.061
No	229 (61.7%)	74 (32.3%)	155 (67.7%)		
**Use of traditional treatment**					
Yes	256 (69.0%)	56 (21.9%)	200 (78.1%)	19.5	**<0.0001**
No	115 (31.0%)	51 (44.3%)	64 (55.7%)		
**Presenting chief complaints**					
Breast lump or mass	328 (88.4%)	86 (26.2%)	242 (73.8%)	16	**<0.0001**
Swelling or lump in armpit	38 (10.2%)	16 (42.1%)	22 (57.9%)		
Painful breast lump/wound	210 (56.6%)	43 (20.5%)	167 (79.5%)		
Other^a^	12 (3.2%)	3 (25.0%)	9 (75.0%)		
**Method of detection of symptoms**					
Accidentally	248 (66.8%)	103 (41.5%)	145 (58.5%)	0.3	0.288
During breast feeding	57 (15.4%)	26 (45.6%)	31 (54.4%)		
During BSE	43 (11.6%)	19 (44.2%)	24 (55.8%)		
Other^b^	23 (6.2%)	12 (52.2%)	11 (47.8%)		
**Time to presentation**					
<3 months	90 (24.3%)	37 (41.1%)	53 (58.9%)	8.7	**0.003**
≥3 months	281 (75.7%)	70 (24.9%)	211 (75.1%)		
**Misdiagnosed at first visit**					
Yes	107 (28.8%)	22 (20.6%)	85 (79.4%)	5.0	0.025
No	264 (71.2%)	85 (32.2%)	179 (67.8%)		

NB.

a Other, Nipple retraction, nipple discharge, skin colour change

b Others, When produces discharge and pain

**Table 3.** Factors associated with advanced-stage diagnosis of breast cancer in north-west Ethiopia, 2020.

Determinant factors	Stage at diagnosis		COR with 95% CI	AOR with 95% CI	*p*-value
	Early stage	Advanced stage			
Age group					
<30	21 (38.2%)	34 (61.8%)	1	1	
30–39	33 (28.0%)	85 (72.0%)	1.6 (0.81, 3.13)	1.4 (0.64, 3.03)	0.404
40–49	21 (22.8%)	71 (77.2%)	**2.1 (1.00, 4.33)**	1.6 (0.73, 3.87)	0.221
50–59	15 (28.3%)	38 (71.7%)	1.6 (0.69, 3.51)	0.9 (0.37, 2.43)	0.924
≥60	17 (32.1%)	36 (67.9%)	1.3 (0.59, 2.89)	0.8 (0.36, 2.18)	0.784
Home residence					
Rural	61 (24.5%)	188 (75.5%)	1.9 (1.17, 2.97)	1.7(1.02, 2.96)	** 0.041**
Urban	46 (37.7%)	76 (62.3%)	1	1	
Painless breast lump					
Yes	64 (39.8%)	97 (60.2%)	1	1	**0.001**
No	43 (20.5%)	167 (79.5%)	2.6 (1.62, 4.06)	2.5 (1.45, 4.13)	
Travel distance					
<5 km	38 (23.3)	125 (76.7)	1	1	**<0.001**
≥5 km	136 (65.4)	72 (34.6)	3.9 (2.43, 6.30)	3.2 (1.72, 5.29)	
Practising BSE					
Yes	28 (62.2%)	17 (37.8%)	1	1	**0. 009**
No	79 (24.2%)	247 (75.8%)	5.2 (2.68, 9.90)	2.9 (1.30, 6.52)	
Use of traditional treatment					
Yes	56 (21.9%)	200 (78.1%)	2.9 (1.78, 4.56)	1.0 (0.58, 1.84)	0.921
No	51 (44.3%)	64 (55.7%)	1	1	
Time to presentation					
<3 months	37 (41.1%)	53 (58.9%)	1	1	**0.021**
≥3 months	70 (24.9%)	211 (75.1%)	2.1 (1.28, 3.47)	1.4 (1.02, 2.37)	
Misdiagnosed at first visit					
Yes	22 (20.6%)	85 (79.4%)	1.8 (1.07, 3.13)	1.9 (1.09, 3.59)	**0.024**
No	85 (32.2%)	179 (67.8%)	1	1	

NB. COR, Crude odds ratio; AOR, Adjusted odds ratio; Bold, Indicates statistically significant associations
